# Celiac Plexus Block to Treat Refractory Feed Induced Dystonia in Children with Severe Neurodisability: A Single Center Case Series

**DOI:** 10.1097/PG9.0000000000000134

**Published:** 2021-10-25

**Authors:** David I. Campbell, Rosalind M. Rabone, Ala Fadilah, Ashok Raghavan, Arun N. Urs, Ayman Eissa, Richard M. Lindley, Santosh Mordekar

**Affiliations:** From the Sheffield Children’s Hospital NHS Foundation Trust UK, Sheffield, United Kingdom.

## Abstract

This observational study describes the procedure technique, safety outcomes, and patient responses to celiac plexus blockade (CPB) in children with severe neurodisability with refractory feed intolerance, feed induced pain or feed induced dystonia (FID).

**Method:**

A review of the pathophysiological response to feeding in children with significant neurodisability and the effect on the neuroenteric system. A 2-stage CT-guided temporary celiac plexus blockade followed by neurolysis technique is described. We compile a case series of 5 patients with life limiting conditions and significant disability undergoing CPB in a single tertiary pediatric hospital.

**Results:**

A total of 10 separate procedures in 5 children were completed. A positive outcome was observed in 3 out of 4 cases of pediatric FID. Two of the three patients on parenteral nutrition had improved feed tolerance postprocedure. All children tolerated the procedure well, no postprocedure complications were documented.

**Conclusions:**

In selected cases, children with life-threatening feed induced dystonia or effective intestinal failure can be safely treated with celiac plexus blockade when other therapies have failed.

## INTRODUCTION

The success story of pediatric medicine is the survival of thousands of children with previously fatal conditions. The great challenge to pediatric medicine is how we continue to care for these same children who are now surviving years later. We are recognizing new complications for old conditions, as children survive into adolescence and beyond. These complications include problems of gut motility and aberrant somatic responses to enteral feeding, including feed induced dystonia (FID).

As part of a working group formulating guidelines for treatment and investigation on this condition, The British Paediatric Neurology Association has offered a case definition of Feed Induced Dystonia as “Enteral feeding repeatedly leading to pain, somatic disturbance (sweating, tachycardia), signs of intestinal dysmotility and dystonic spasms that settles with fasting and is not due to gastroesophageal reflux disease or anatomical abnormalities of the GI tract” (1).

FID occurs in children with severe central nervous system (CNS) pathologies often associated with dystonias. The episodes of dystonia may be so severe, leading to status dystonicus, and be accompanied by rhabdomyolysis, hip dislocation, and pneumatosis coli. Currently, the treatment of FID is aimed at reducing systemic doses of antidystonia and antispasticity drugs, resting the gut before gradual introduction of enteral feeds. The understanding of how the scenario arises, or whether it is some form of extreme drug side effect in the vulnerable patient is unknown, the field is new and hence likely to change with time and future research. We describe our experiences, within a tertiary pediatric children’s hospital in the United Kingdom, of using celiac plexus blockade in the treatment of feed induced dystonia and refractory feed intolerance and abdominal pain after reviewing the pathophysiological response to feeding in children with significant neurodisabilities. All affected children had complex CNS pathology associated with dystonia, were nonverbal and nonmobile. It should be noted that a totally denervated bowel (small bowel graft) is painless but functional. The underlying problem of feed associated pain is related to the consequences of abnormal processing of afferent sensory signals from the foregut.

### Afferent Innervation of the GI Tract

Children with feed induced dystonia, begin to experience pain and show signs of discomfort within seconds of enteral feed commencing. The speed of response suggests, that the somatic responses to enteral feeds is likely to be neuronal sensory (afferent) signals. The foregut includes the stomach and duodenum and is innervated by the vagal and thoraco-lumbar vagal afferent fibers (derived from the left and right branches of the vagus nerve) (2). The right branch of the nodose ganglion innervates the duodenum down to the terminal ileum, the third and fourth parts of the duodenum are innervated by fibers running to the left nodose ganglion (3). Sensory fibers run from the small bowel mucosa, where both glial cells in the lamina propria and submucosa and sensory nerve endings interact with a variety of neuroendocrine, nutrient, and immune signals to send neural signals that lead to motility adaptations (4). Enteric neurons use a plethora of neurotransmitters including adrenergic, cholinergic dopaminergic, nitrous oxide, 5-HT, and adenosine. Sensory fibers run through the celiac plexus to the dorsal route ganglia and then in the lateral spinothalamic tracts to the nucleus tractus solitaris in the brain stem. From the NTS fibers synapse with centers in the thalamus, amygdala, Vth motor nucleus, nucleus ambiguous, prefrontal sensory cortex, cingulate gyrus, insula, and other parts of the limbic system via third and fourth order neurons (5,6). It is these distant, and often overlooked structures within gastroenterology, where normally subconscious visceral sensory signals lead to conscious and measurable responses. Spinothalamic tracts carry pain signals, while thalamic and brain stem nuclei control a variety of autonomic functions (7). Damage to interconnecting or thalamic/brainstem nuclei could lead to failed homeostatic control of feedback loops that regulate gut motility and secretion.

When enteral feed leads to repeated episodes of severe pain or life-threatening status dystonicus or intestinal failure due to accompanying dysmotile gut failure, the option of a sensory blockade of the abnormally innervated GI tract is very attractive, in order to achieve full enteral feeding. Injection of drugs that would temporarily or permanently inhibit stimulatory sensory signals into the celiac plexus could potentially achieve this aim.

## METHODS

### Technique: CT-guided Posterior Para Aortic Celiac Plexus Block

Informed, written consent was obtained from the families of all the patient’s undergoing CPB before procedure by the lead clinician after lengthy outpatient counseling. Ethics approval was not sought as CPB is an established procedure in oncological and palliative care settings for refractory abdominal pain. Each family consented to the reporting of the CPB and outcome in this case series either in writing or verbally.

Patients were all recruited from the wards of a single UK center between May 2013 and September 2018 and were sequentially recruited. The study design is a case series. Details of patient neurological diagnoses and GI pathologies are given in Table 1. Patients were identified from the gastroenterology and neurology services as children who had severe neurodevelopmental pathology but also had or were looking likely to require PN.

**TABLE 1.** Clinical details of cases undergoing CPB

Case no.	Sex/age	Diagnoses	GI diagnoses	Feeding device	PN requirement
1	F/9	Trisomy 3 Monosomy 5	Chronic intestinal pseudo obstruction Chronic pancreatitis Cholecystitis with cholecystectomy Pneumatosis coli Colostomy Fundoplication	PEG PEG-J	Yes Home PN
2	F/17	Leigh encephalopathy Epilepsy	Foregut dysmotility	PEGJ	No
3	F/6	Chromosome 2q deletion Epilepsy Neuropathic bladder	Foregut dysmotility	PEGJ	Yes Home PN
4	F/15	Traumatic brain injury Spastic tetraparesis Epilepsy Intrathecal baclofen pump,	GORD Fundoplication	PEGJ	Yes Home PN
5	F/7	Four limb cerebral palsy Ex-preterm Periventricular leukomalacia Lower limb infarction with amputation	GORD Fundoplication	PEGJ	Yes Short term

CPB = celiac plexus blockade.

CT-guided celiac plexus block was performed under general anesthesia. All prior CT abdominal studies were evaluated in planning the procedure. An updated contrast CT would be done if no imaging was available within 6 months, before a celiac plexus block. The child was placed in a prone position after general anesthesia with support under the abdomen to increase the thoracic kyphosis. This approach facilitates a bilateral posterior antecrural approach, hence avoiding the posterior costophrenic sulcus, which would increase the risk of pneumothorax.

**FIGURE 1.**
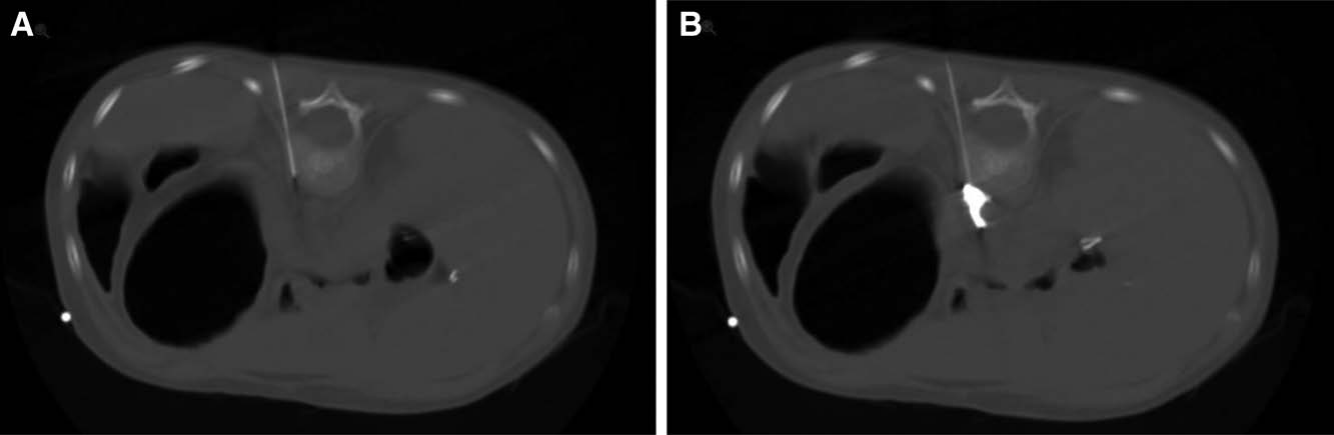
CT-guided posterior lumbar approach to the celiac plexus. A) Direction of approach to the anterior margin of L1 body. B) Contrast dispersion to confirm correct placement of 22G needle in retroperitoneum.

The origin of the celiac axis from the abdominal aorta is identified from the contrast CT, and the shortest and least complicated route to the celiac plexus is determined. Aseptic technique was used. A 22G beveled spinal needle was advanced under CT guidance from posterior to anterior toward the ventral surface of the vertebral body (Fig. 1A). Once the vertebral body had been contacted the spinal needle was advanced by about 0.5 to 1 cm at a time further into the prevertebral fascial plane. Once the needle was in the desired position, the placement was confirmed by the spread of contrast under CT visualization (Fig. 1B). A diagnostic block with local aesthetic or a therapeutic neurolytic block (such as 100% alcohol) can be used. A unilateral block on the left-side was shown to be reliable. A bilateral block could be performed if the unilateral block does not provide sufficient results.

Pain scores before and after the procedure were borrowed from the visual analogue scale (VAS), scored by parents from 48 hours to 72 hours post procedure and compared with the mean score for the 2 weeks leading up to the CPB.

Procedural serious complications, particularly pneumothorax and retroperitoneal hematoma/pancreatitis were screened for with nursing observations of temperature, respiratory and heart rate, plus oxygen saturations. Post blockade biochemistry (amylase and lipase) was checked at 24–48 hours to ensure no pancreatitis.

## RESULTS

Five cases, all female aged 7–17 years of age underwent CT-guided posterior approach celiac plexus blockade (Table 1). The clinic letters and electronic patient notes were retrospectively reviewed for all patients.

All had severe neurodisability either due to perinatal asphyxia, genetic/metabolic disease or head trauma. All had a history of feed induced abdominal pain, 3 had a history of feed induced dystonia. All had been investigated for current reflux disease and anatomical problems with the GI tract excluded by barium meal. Four out of the five patients were scored with a visual analogue scale for pain (VAS).

### Case 1

Case 1 underwent 5 separate CPBs (the first being diagnostic, the subsequent being neurolytic. Two neurolytic blocks were done on each side, with temporary improvements each time). A mean reduction in VAS of 48.2 mm (range 34–57 mm) and a mean increase in enteral feeds of 13.6 mL/h (range 6–24) on each occasion. There was reduced oramorph requirement after 3/5 procedures (approximately 30–50% of the preblockade levels). It became evident that the effect was not maintained using the method of pharmacological blockade. Further procedures were deemed futile.

### Case 2

Case 2 had a temporary and then a permanent CPB within 3 months of the first diagnostic CPB. Sixty-five millimeter improvement on the VAS pain score was achieved, an increase in enteral feeds of 40 mL/h after the first block and maintained for 6 months after the neurolytic block. Analgesia was successfully weaned off completely. Periods of IV fluids were used to settle episodes of dystonia, and these were not required for 6 months after the neurolytic (second) CPB. On both occasions, marked improvements in pain feed tolerance and general well-being, there was accompanying loose stools up to 3 times a day. The loose stools were well tolerated and general improvements in well being were achieved by the absence of pain and improved nutrition. When the diarrhoea resolved, the pain and feed intolerance returned.

### Case 3

Case 3 required PN due to life threatening episodes of status dystonicus. Following CPB, she had an improvement in VAS pain score of 84 mm, and a change in oramorph requirement from 4 times daily to 2–3 times per week (daily equivalent morphine use fell from 1 mg/kg/day to approximately 0.08 mg/kg/day). Unfortunately, though feed tolerance increased, it was not to the point of coming off PN completely. Stools were transiently loose following the CPB.

### Case 4

Case 4 had a single CPB with a reduction in VAS of 2 mm, no change in analgesia or enteral feeds. This was regarded as a negative response or failed procedure.

### Case 5

In patient observations gave conflicting impressions on how much pain the child was experiencing. At times parent and professionals agreed on the presence of pain, at times there was not a concordant view. There were no episodes of feed dystonia but feed associated pain. In the end, it was decided to offer a CPB with a detailed consent to the possibility of a failed procedure. There was no perceived benefit from the CPB by either parent or professional. A neurolytic CPB was not performed, only a single temporary nerve block.

All children tolerated the procedure well. There were no complications after a total of 10 separate procedures.

## DISCUSSION

A total of 10 CPB procedures on 5 patients have been undertaken at a single centre. Each child had limited life expectancy and standard methods of pain control, including input from the pain service at a UK children’s teaching hospital, had failed to control painful dystonias. Feed intolerance will lead to death by malnutrition and two of the children had been started on PN with further associated risks to life. It was felt on balance that a temporary CPB, when all other avenues had failed, would be a relatively safe, compassionate therapy.

Parents consented to the procedure after a discussion on the possible risks and benefits. The quality of life with standard palliative or analgesic/dystonia therapies had been judged by the parents to be inadequate. Critical to the assessment of quality of life is the assessment of pain. We opted for a ready to use scale (VAS), but adapted this from self to observer reported. This is commonly done in the care of children with severe neurodisability, but the VAS system is not validated for observer scoring. This is a weakness to the reported case series, but does provide some quantification of “how effective” the intervention has been.

The patients presented in this case series and the clinical contexts is vital, to understand why a CPB was offered. FID is extremely painful, with severe somatic disturbances brought on by enteral feeding (tachycardia, sweating, crying, and dystonias with fever). The alternative treatments with PN, central vein catheterisation bring with them risks of sepsis, liver disease, pericardial effusions, thrombosis, and so on. The risks of a CPB such as retroperitoneal hematoma and pancreatitis are also serious. The consent process has to be detailed and well documented. The goals of pain relief and establishment of enteral feeds, when other therapies have failed, in severely neurologically disabled children leads to consideration of new procedures such as a CPB. These children had previously had a better quality of life, before the development of FID severely undermining that quality of life. Alternative explanations for feed associated pain, leading to feed associated pain are always sought (GORD, constipation, hip dislocation, urinary retention, and so on). In particular pneumatosis coli is screened for with an abdominal radiograph (8). Due diligence was applied before a patient underwent a CPB in terms of standard operative risks such as cardiopulmonary health, or bleeding disorders/risks. Patients with haemophilia or platelet disorders would present a different risk benefit ratio.

In all the children, dystonias were seen to settle with 24 hours of gut rest on intravenous fluids. The periods of being settled enabled, where possible, the reduction of high dose dystonia medications, to moderate doses. Repeated challenges with enteral feeds, despite medication reduction lead to recurrence of FID.

The procedure was offered to parents on the basis of a theoretical mechanism of disease, based more on careful, repeated, clinical observations. The immediate recurrence of dystonia following the introduction of enteral feeds in these children, strongly suggested aberrant afferent autonomic signals.

The origin of those signals had to be the foregut due to the immediacy of response with gastric or jejunal feeding. The sensory innervation of the foregut includes the second loop of jejunum and this is not possible to bypass without a deep feeding jejunostomy, which themselves are subject to greater risks of volvulus, intussusceptions, and fistula leakage.

Inserting deep feeding jejunostomies with a combined enteroscopic/laparoscopic approach is far more invasive than the CPB that we proposed.

On the basis of two of the three successful cases, the association with intestinal hurry, suggests this is a potential marker of procedure success.

When focusing on the issue of feed induced dystonia, rather than feed associated abdominal pains, a positive outcome was observed in 3 out of 4 cases, even if only temporary. Dystonic spasms that start within minutes are dramatic and painful. For situations where pain rather than dystonia with pain, there is a risk that over reporting of pain by carers (and under documenting pain by professionals) is possible, leading to inevitable failure of the CPB to produce a positive outcome. The parents with severely disabled children, on the one hand and doctors keen to help, on the other, can lead to a CPB being performed, with little chance of positive response. On this matter, either no procedure or a temporary nerve block, rather than a neurolytic block is an option to consider after the appropriate discussion and consent. It is worth noting that methods of assessing pain in a nonverbal child are necessarily problematic and an area of active development and research (9). We would like to highlight the difficulties in both underestimating a child’s level of pain (and possibly denying them pain relief) or overestimating and potentially putting the child through an unnecessary procedure. Developments in pain assessment in the nonverbal child would be of great help in these situations.

## CONCLUSIONS

Children with life-threatening feed induced dystonia or effective intestinal failure (as a result of the effects of enteral feed on tone and movement), can in selected cases be treated with CPB, when other therapies have failed. A 2-stage procedure of temporary blockade followed by neurolysis is proposed to ensure a high chance of patient benefit but minimising patient risk.
